# Single-Incision Thoracoscopic Surgery using Spinal Needle Anchoring

**DOI:** 10.1055/a-2652-7894

**Published:** 2025-07-31

**Authors:** Jongbae Son, Dae Hyun Kim, Sang-Ho Cho

**Affiliations:** 1 Department of Thoracic and Cardiovascular Surgery, Kyung Hee University Hospital at Gangdong, Gangdong-gu, Seoul, Korea; 2 Department of Thoracic and Cardiovascular Surgery, Kyung Hee University College of Medicine, Dongdaemun-gu, Korea

**Keywords:** thoracoscopy/VATS, pneumothorax, minimally invasive surgery, single-incision thoracoscopic surgery

## Abstract

Single-incision thoracoscopic surgery (SITS) for primary spontaneous pneumothorax offers advantages over multiport video-assisted thoracoscopic surgery, but lesion retraction remains challenging. We describe a modified SITS technique using spinal needle anchoring for precise lung stabilization. A bent spinal needle inserted through an intercostal space mimics a second-port grasper, enabling multiple re-hooking maneuvers for optimal lesion alignment. This technique allows for a smaller incision, minimizes instrument crowding, and reduces postoperative pain. The needle insertion site leaves no visible scar. Additionally, it is beneficial in cases with multiple bullae or challenging lung anatomy. **Spinal needle anchoring may represent a simple and effective modification of the SITS technique** .

## Introduction

Video-assisted thoracoscopic surgery (VATS) is widely used in the surgical treatment of primary spontaneous pneumothorax (PSP) and is classically performed through several ports. Single-incision thoracoscopic surgery (SITS) for wedge pulmonary resection was first introduced in the early 2000s. Over time, efforts have been made to further minimize instrument use at the incision site, while modified SITS techniques have also been developed to enhance lesion retraction. We present a modified SITS technique for PSP that utilizes spinal needle anchoring.

## Technique Description

**Video 1** Skin incision to insertion of the spinal needle.


**Video 2** Multiple re-hooking maneuvers with a single needle.


**Video 3** Wedge resection of multiple bullae using two spinal needles simultaneously.


**Video 4** Multiple re-hooking maneuvers along the fissure margin.


**Fig. 1:**
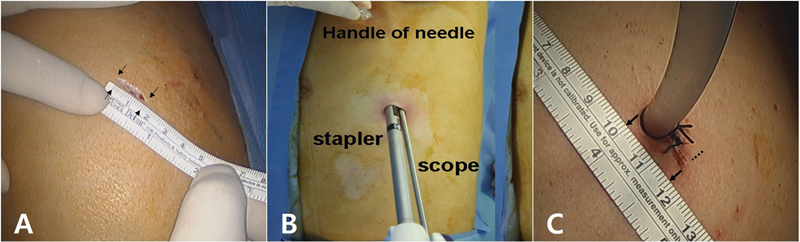
Surgical procedure and incision sites. **(A)** A 1.5-cm incision along the mid-axillary line (arrows). **(B)** Intraoperative view showing the single port with the thoracoscope and stapler. **(C)** Final wound after 20-Fr tube insertion, measuring 1.5 cm (arrows).

SITS using spinal needle anchoring involves inserting a spinal needle through a separate intercostal space—distinct from the single-incision site, to anchor the lung within the thoracic cavity. This procedure was originally developed and modified by Dr. Dae Hyun Kim. Surgery was performed under general anesthesia with the patient in the lateral decubitus position. A 1.5-cm skin incision was made along the mid-axillary line at the level of the fifth intercostal space ( Fig.1A ). If a chest tube was already in place, the existing insertion site was utilized as the single port for SITS. A 5-mm, 30-degree thoracoscope was inserted after placing a wound retractor and secured to one side of the incision, allowing for the introduction of a grasping instrument to evaluate the number, location, and condition of the lesions, such as bullae or blebs. Subsequently, the distal 2 cm of a 20-gauge spinal needle was bent at a 90-degree angle and inserted into the thoracic cavity, typically through the anterior axillary line at the second intercostal space ( Video 1 , available in the online version only). However, the insertion site may vary depending on the location of the lung lesion, with the needle placed at the corresponding intercostal space as needed.

**Fig. 2:**
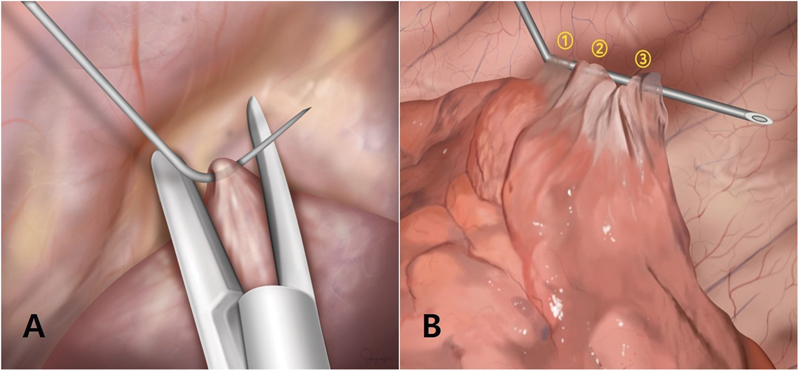
Spinal needle anchoring technique for lung retraction. **(A)** Illustration demonstrating the spinal needle anchoring technique. **(B)** Multiple re-hooking maneuvers using a single needle.

After inserting the spinal needle, the affected lung tissue was grasped using a grasping instrument and carefully hooked onto the needle ( Fig. 2A ). Although a single hooking approach can be used, multiple adjustments and re-hooking maneuvers were performed as needed to optimize the tension and alignment of the lesion with the planned resection line ( Fig. 2B and Video 2 , available in the online version only). The lung, hooked onto the spinal needle, was repositioned by rotating or adjusting the needle vertically under the guidance of the grasping instrument to ensure proper orientation for stapling or cutting. Once the lesion was adequately stabilized with the spinal needle, an endoscopic stapler (Medtronic Endo-GIA™) was introduced through the single port ( Fig. 1B ). The stapler was positioned along the planned resection line, and the lung tissue containing the lesion was excised ( Fig. 2A and Video 2 , available in the online version only). After completing the wedge resection, an air leak assessment was performed using an underwater test. To minimize the risk of recurrence, the resection site and the surrounding lung parenchyma were reinforced with polyglycolic acid felt (Neoveil®) and fibrin glue. A 20-Fr chest tube was inserted through the incision site ( Fig. 1C ), and the spinal needle insertion site was managed with a simple dressing.

When the base of a bulla is broad or when multiple adjacent bullae require a larger resection area, two or more spinal needles can be placed simultaneously at different sites to enable a more extensive wedge resection ( Video 3 , available in the online version only). In some cases, multiple bullae are distributed along the major or minor fissure, specifically along the peripheral fissure margin. In such situations, employing multiple re-hooking maneuvers allows for effective wedge resection while preserving the natural lung contour and avoiding deformation ( Video 4 , available in the online version only).

## Discussion

SITS was introduced in the early 2000s for procedures like sympathectomy. In 2004, Rocco et al. expanded their application to wedge resections, and by 2010, Gonzalez et al. successfully performed lobectomies. 1 2 Over subsequent decades, SITS has advanced significantly, driven by surgical techniques, instrumentation, and clinical experience. SITS for major anatomical lung resection in lung cancer has been adopted by many centers but remains an emerging approach due to challenges in vessel, bronchus, and mediastinal lymph node management, requiring precise dissection and secure handling. 3 For minor lung resections, such as PSP or small peripheral lesions, SITS is considered safe and feasible. It provides advantages over multiport VATS, including minimal scarring, reduced postoperative pain, less chest wall paresthesia, and shorter hospitalization. Its morbidity is comparable to multiport VATS, and its recurrence rate aligns with the best outcomes reported for multiport VATS or thoracotomy. 4 However, SITS for wedge resection has not been widely accepted as standard due to its limitations, including instrument collision, restricted field of view, and the need for a slightly longer single incision to accommodate multiple instruments. To overcome these limitations, a laparoscopic port (originally developed for abdominal procedures) and articulated grasping instruments have been adapted for SITS. 5 Additionally, various techniques have been introduced to improve SITS feasibility, including methods that facilitate lesion traction using a percutaneous loop retractor, hook wire, or anchoring suture. 6 7 8

Unlike previous anchoring techniques, we utilized a 90-mm spinal needle to hook the lesion and retract the lung in an appropriate direction for stabilization. This technique offers several advantages, as the spinal needle functions similarly to a grasping instrument used in a second port in multiport VATS. First, multiple re-hooking maneuvers with a single needle allow for precise traction in the optimal direction, facilitating accurate resection while preserving lung contour and preventing deformation after stapling. Second, since the lesion's location can vary beyond the upper lobe apex, the needle insertion site can be selected to correspond to the lesion's position and easily repositioned as needed. Third, in patients with bullous lung disease or multiple large bullae, two or more spinal needles can be used simultaneously for a broader wedge resection, enhancing procedural feasibility. Additionally, the needle insertion site leaves no visible scar, and postoperative pain is minimal. Finally, stable lung fixation through spinal needle traction minimizes stapler movement, allowing a reduction in skin incision size to 1.5 cm. This technique has been previously applied in 139 patients at our institution with favorable outcomes. No conversion to multiport VATS was required, and the recurrence rate was low (2.16%) over a mean follow-up of more than 7 years. Postoperative complications were not observed, supporting its feasibility and safety of this approach. 9 However, this technique has some limitations. SITS using spinal needle anchoring offers a limited ability to explore the lung parenchyma. Therefore, it is not suitable for wedge resection of non-visible or deep-seated pulmonary nodules. In obese patients, needle handling may be challenging, but this can be improved by using a more rigid 19-gauge needle bent at 90 degrees over a 3-cm segment.

## Conclusion

SITS for PSP utilizing a spinal needle as a substitute for the grasper is a safe and effective technique that can be performed through a minimal incision. This approach can facilitate precise resection while preserving lung integrity, making it a valuable option even in anatomically challenging cases.

## References

[1] Rocco G Martin-Ucar A Passera E Uniportal VATS wedge pulmonary resections Ann Thorac Surg 2004 77 02 726 728 14759479 10.1016/S0003-4975(03)01219-0

[2] Gonzalez D Paradela M Garcia J Dela Torre M Single-port video-assisted thoracoscopic lobectomy Interact Cardiovasc Thorac Surg 2011 12 03 514 515 21131682 10.1510/icvts.2010.256222

[3] Vieira A Bourdages-Pageau E Kennedy K Ugalde P A The learning curve on uniportal video-assisted thoracic surgery: An analysis of proficiency J Thorac Cardiovasc Surg 2020 159 06 2487 249500 31926696 10.1016/j.jtcvs.2019.11.006

[4] Masmoudi H Etienne H Sylvestre R Three hundred fifty-one patients with pneumothorax undergoing uniportal (single port) video-assisted thoracic surgery Ann Thorac Surg 2017 104 01 254 260 28410634 10.1016/j.athoracsur.2017.01.054

[5] Assouad J Vignes S Nakad J Grunenwald D Single incision video-assisted thoracic surgery using a laparoscopic port Ann Thorac Surg 2011 91 06 2020 2021, author reply 2021 21620013 10.1016/j.athoracsur.2010.11.050

[6] Mogi A Yamaki E Kosaka T Asao T Kuwano H Thoracoscopic wedge resection through a single incision using a thin puncture device Ann Thorac Cardiovasc Surg 2014 20 03 198 201 23666247 10.5761/atcs.oa.13.02278

[7] Son B S Kim D H Lee S K Kim C W Small single-incision thoracoscopic surgery using an anchoring suture in patients with primary spontaneous pneumothorax: A safe and feasible procedure Ann Thorac Surg 2015 100 04 1224 1229 26212513 10.1016/j.athoracsur.2015.04.095

[8] Chong Y Cho H J Kang S K Outcomes of the Tower Crane Technique with a 15-mm trocar in primary spontaneous pneumothorax Korean J Thorac Cardiovasc Surg 2016 49 02 80 84 27066430 10.5090/kjtcs.2016.49.2.80 PMC4825907

[9] Lee S H Lee S G Cho S H Song J W Kim D H Outcomes of single-incision thoracoscopic surgery using the spinal needle anchoring technique for primary spontaneous pneumothorax J Chest Surg 2022 55 01 44 48 35115421 10.5090/jcs.21.132 PMC8824646

